# In Vitro Gamete Production in Mammals: Decades of Pioneering Approaches in Germ Cell and Stem Cell Biology Supporting Innovative Discovery and Expansion of Human Reproductive Potential

**DOI:** 10.1002/mrd.70072

**Published:** 2025-12-11

**Authors:** Keith E. Latham

**Affiliations:** ^1^ Department of Animal Science Michigan State University East Lansing Michigan USA; ^2^ Department of Obstetrics, Gynecology and Reproductive Biology Michigan State University East Lansing Michigan USA

**Keywords:** artificial gametes, assisted reproduction technologies, germ cells, in vitro gametogenesis, stem cells

## Abstract

During recent decades a myriad of approaches have emerged for generating surrogate gametes or achieving in vitro gametogenesis (IVG). The most recent approaches to IVG include the development of increasingly complex culture systems, use of companion cells combined with germ line cells in two‐ or three‐dimensional configurations, derivation of germ line‐like cells from pluripotent stem cells, and genetic and chromosomal manipulations of pluripotent stem cells to enable the production of bi‐maternal and bi‐paternal offspring. This review summarizes the major approaches in IVG, the challenges remaining, and considers the significance of these technologies within the context of departure from exclusively dioecious sexual reproduction for the human species.

## 1 Introduction

Sexual reproduction has existed for about two billion years, and occurs in about 99.99% of all eukaryotes (Otto 2008), with most contemporary parthenogenetic or asexually reproducing eukaryotes having arisen recently through loss of the sexual reproduction mode (Charlesworth 2006). The basis for the evolutionary rise and predominance of sexual reproduction in the face of significant costs has been the subject of considerable study (Charlesworth 2006; Goodenough and Heitman 2014; Otto 2008; Otto and Gerstein 2008).

Essential features of sexual reproduction that must be met to realize its evolutionary benefits include (1) alternation of diploid and haploid phases through cycles of cell–cell fusion (either as distinct haploid/diploid phases of life seen in unicellular organisms, or as haploid gamete/diploid organismal portions of the life cycle in multicellular species), (2) meiosis, during which chromosome replication, pairing, recombination and segregation occur leading to genetically recombined genomes within gametes, (3) the production of gametes that ensure appropriate transmission of organelles and genomes, and (4) mechanisms related to mating‐type control of cell–cell fusion (Charlesworth 2006; Goodenough and Heitman 2014). The fulfillment of these features is enabled by complex cellular and molecular mechanisms driven by an equally complex set of genes that confer both shared and increasingly divergent functions that have emerged to enable different modes of sexual reproduction. Collectively, these functions provide an essential continuity of life for the vast majority of multicellular eukaryotes.

With these evolutionary thoughts in mind, it is striking to witness the recent emergence of new reproductive technologies that enable the in vitro production of gametes and embryos, and even the production of sperm or oocytes from either males or females. Mammals naturally exhibit exclusively dioecious sexual reproduction. Experimental parthenogenesis, cloning by nuclear transfer and other laboratory methods previously provided approaches for enabling asexual modes of reproduction in laboratory mammals. The new technologies of in vitro production of “artificial” gametes have opened the door for further exceptions. Reproduction using artificial gametes would comprise a uniquely *Homo sapiens* innovation that would not only provide for novel treatments of diseases, infertility, and reproductive senescence, but also place our species at the threshold of realizing new modes (e.g., bi‐maternal or bi‐paternal) of sexual reproduction. This review presents a summary of different approaches to in vitro gamete production, the value, barriers and concerns with the current methods, and the potential for these methods to interface with other emerging methods to achieve a diverse set of reproductive goals.

## 2 Approaches to In Vitro Gametogenesis

**Figure 1:**
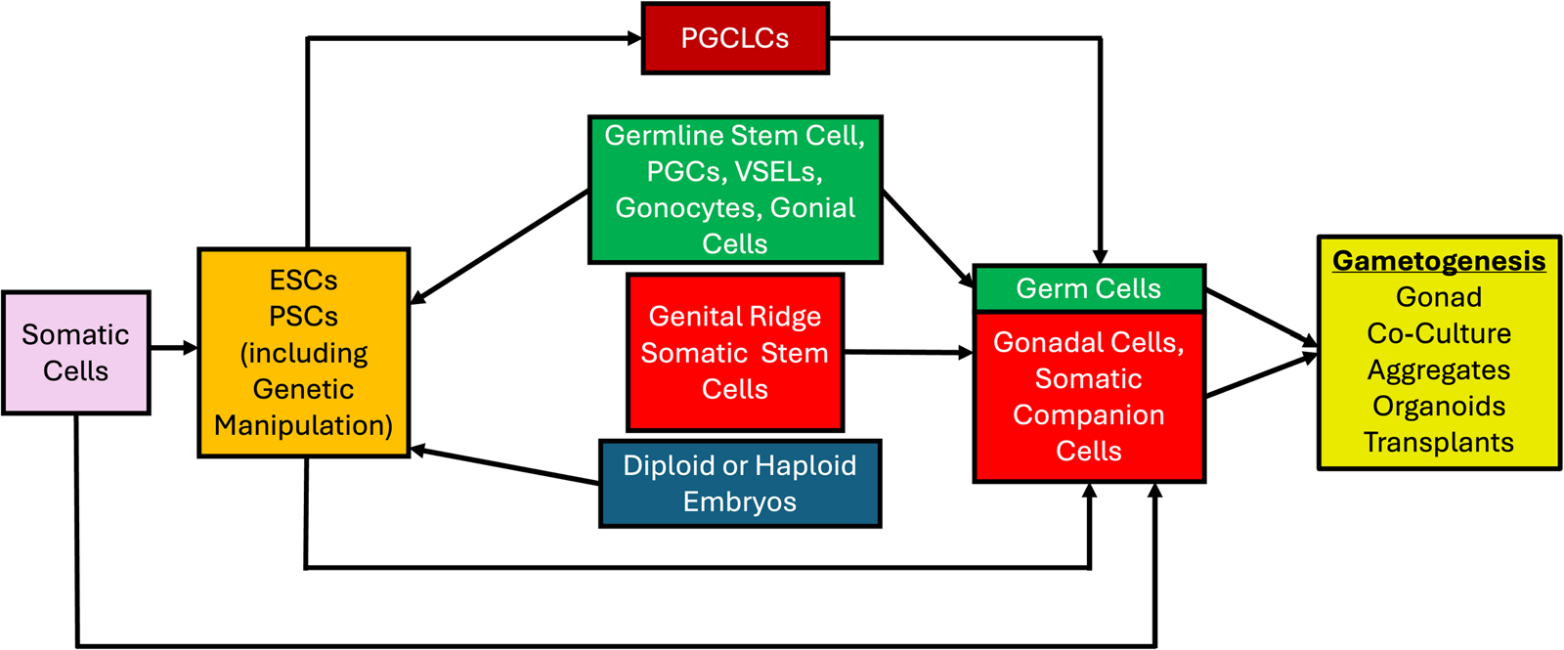
Summary of cell sources and cell conversions used for in vitro gametogenesis, and relationship to in vivo gametogenesis. The conversions diagrammed here are in principle achievable in any species, although specific methodologies may differ across species. Germline and somatic components in the embryo (red and green) work together to form gonads. Germ line cells differentiate and undergo gametogenesis in gonads or via other IVG modes (yellow box) with the support of companion somatic cells. Germline, genital ridge, and adult gonadal somatic cells (e.g., VSELs) can be used for IVG. Haploid and diploid embryos and somatic cells can be used to derive embryonic stem cells (ESCs) and pluripotent stem cells (PSCs). The ESCs and PSCs can be used to derive PGCLCs and companion cells for IVG by multiple modes (yellow box), and haploid cells can be used as surrogate gametes in semi‐cloning methods to generate embryos. Some somatic cells can also be used as companion cells. Somatic stem cells could in theory be used for IVG but more likely success is via the generation of primordial germ cell like cells (PGCLCs).

**Figure 2:**
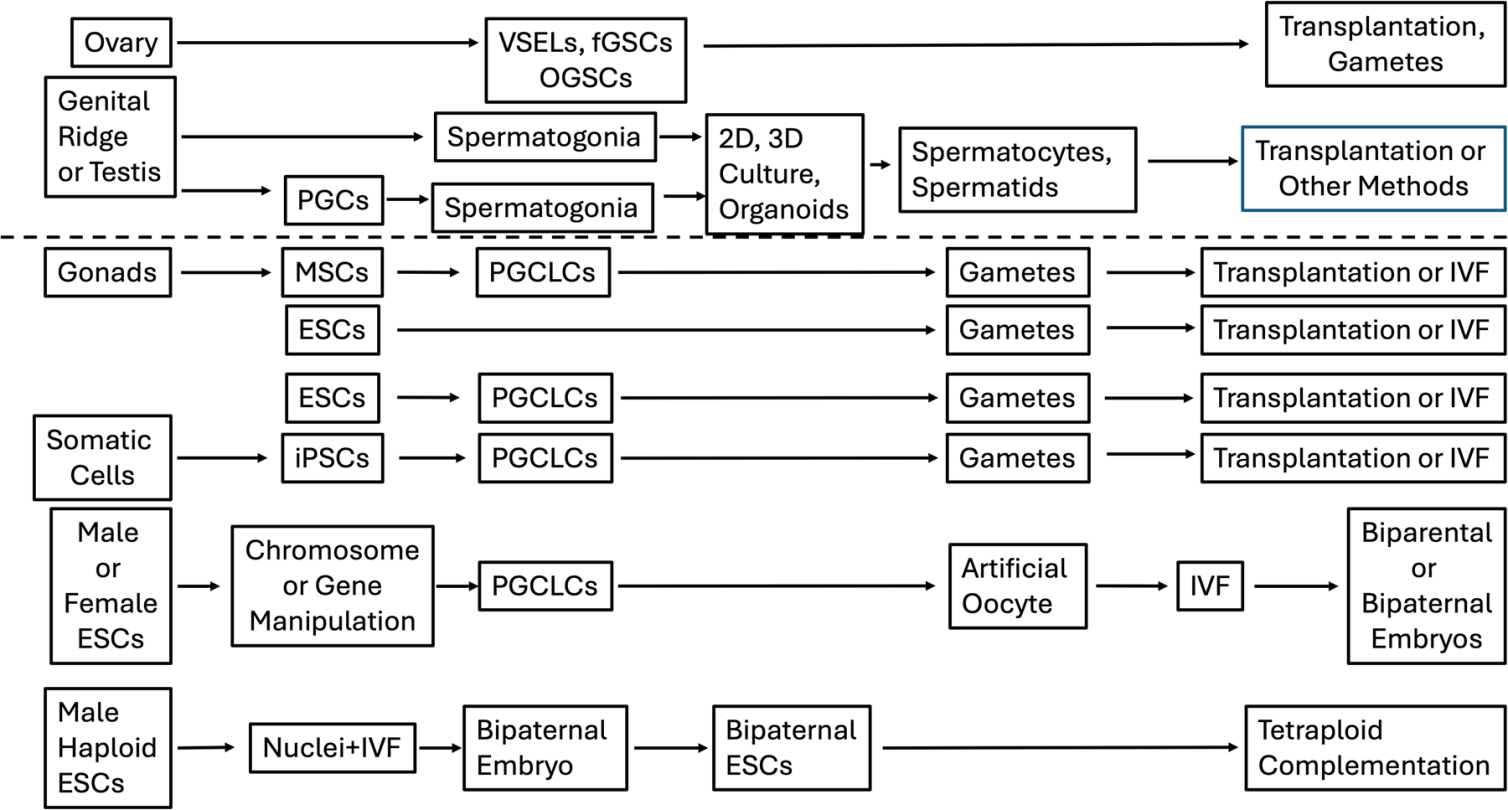
Examples of in vitro strategies for producing gametogenic cells or gametes. The strategies diagrammed here are in principle achievable in any species, although specific methodologies may differ across species. Applications involving genetic manipulations could be problematic for use in humans. Two general strategies pursue IVG by using germ line cells (above dotted line) or using isolated stem cells or pluripotent stem cells (below dotted line) including mesenchymal stem cells (MSCs), embryonic stem cells (ESCs), and induced pluripotent stem cells (PSCs). For the former strategies, gametogenic cells are isolated and expanded in vitro, and subsequently produce gametes using various transplantation methods. For some processes, production of gametes or gametogenic cells is achieved in vitro using advanced culture systems or organoids followed by transplantation or IVF. For strategies using stem cells, gametogenic cells or gametes are produced in vitro from mesenchymal or pluripotent stem cells and used directly for IVF (e.g., IVG oocytes) or subjected to transplantation (e.g., sperm production). The two bottom strategies are from landmark mouse studies conducted by Murakami et al. (2023) and Li et al. (2025). IVF, in vitro fertilization; OGSCs, ovarian germ stem cells; PGCLCs, primordial germ cell‐like cells; PGCs, primordial germ cells; VSELs, very small embryonic‐like stem cells.

Methods of in vitro gametogenesis (IVG) can use a wide variety of cell types and sources (Figure 1) and can be divided into two broad types of strategies (Figure 2). The first of these strategies, and the earliest method applied, derives gametes from germ cell lineage cells, including a range of methods such as germ cell development in gonadal tissue or genital ridge explants, in vitro development of oocytes in isolated ovarian follicles, and differentiation of spermatogonial cells. The second overall strategy for IVG involves approaches using a variety of other stem cell types, including mesenchymal stem cells isolated from gonads, other somatic tissue‐derived stem cells, embryonic stem cells, or induced pluripotent stem cells.

### 2.1 In Vitro Gametogenesis Using Germ Lineage Cells

An early key objective for the field was to establish an in vitro model for studying spermatogenesis and oogenesis. The overall goal was to recapitulate the process from primordial germ cell to functional gamete, so that a wide range of experimental studies could be conducted to study essential molecular and cellular transitions and mechanisms controlling the process, as well as testing impacts of various biological agents or environmental factors (toxins, hormones, nutrients, etc.) on gametogenesis. Many prominent and distinguished pioneering studies were conducted.

#### 2.1.1 Spermatogenesis

Studies pursuing in vitro spermatogenesis from insects (Goldschmidt 1915) and cultured newborn or adult mammalian testis tissues or cells date back over 100 years (Komeya et al. 2018), with numerous sequential advances using diverse species such as rabbit, guinea pig, rat, and mouse (Boitani et al. 1993; Chubb et al. 1978; Feng et al. 2023; Jordan et al. 1961; Libbus and Schuetz 1979; Nagao 1989; Parvinen et al. 1983; Steinberger et al. 1964; Ziparo et al. 1980). Komeya et al. (2018) provide an instructive history of the field and discussion of challenges, obstacles and major advances (Komeya et al. 2018). They describe interesting species differences in success, and also review the remarkable progression from plasma clots, to gas–liquid interphase, to use of defined media employing purified culture components and nutrients and other components identified as critical, to seminiferous tubule culture systems, to combinations of spermatogonial stem cells and testis pieces in organ culture, to the hanging drop method, the agarose gel organ culture system, the development of microfluidic culture systems, coculture systems combining somatic and spermatogonial cells, and two‐ or three‐dimensional culture systems. Exciting innovations in these and other directions have continued (Bashiri et al. 2023; Diao et al. 2022; Ibtisham et al. 2021; Jabari et al. 2020; Richer et al. 2024). Several of the systems have overcome the in vitro pachytene spermatocyte stage block to yield spermatids and complete in vitro spermatogenesis in mice, after which sperm were used to produce viable progeny, and improved culture of seminiferous tubule segments have allowed discovery of ways to enhance spermatogenesis in vitro (Feng et al. 2023; Hakovirta et al. 1999; Komeya et al. 2018; Sato et al. 2011). Additionally, porcine gonocytes were purified from neonatal testis using a selective binding compound called dolichos biflorus agglutinin (DBA), and then sustained in culture for 7 days (Goel et al. 2007), demonstrating potential innovative applications of new methods in other species.

Primordial germ cells (PGCs) isolated from embryos provided additional opportunities for IVG. Early studies of mouse primordial germ cells (PGCs) in embryos (Chiquoine 1954; McLaren 1983) led to early efforts to culture PGCs (De Felici 2001; De Felici et al. 1992; De Felici and McLaren 1983). Studies in mouse and other species led to PGC purification (e.g. (Pesce and De Felici 1995)), identification of essential factors to allow long‐term PGC culture (De Felici and Dolci 1991; De Felici and Pesce 1994; Dolci et al. 1993; Farini et al. 2005; Pesce et al. 1993; Resnick et al. 1992), establishment of PGC cell lines known as embryonic germ cells (EGs) (Kakegawa et al. 2008; Ledda et al. 2010; Petkov et al. 2011; Stewart et al. 1994), and the ability to induce PGCs to generate oocytes and sperm after transplantation (Matoba and Ogura 2011). Although in the male PGCs normally form spermatogenic cells, they can be induced to pluripotency in vitro, and in vivo they can form teratomas (Kee et al. 2010; Kimura and Nakano 2011), illustrating striking PGC developmental potentials. Gonocytes derived from PGCs have more restricted growth and survival requirements compared to PGCs [e.g., requirements for companion Sertoli cells, hormones, and growth factors; (Meehan et al. 2000; Nagano et al. 2003; Orth and McGuinness 1991; Van Dissel‐Emiliani et al. 1996)], and give rise to spermatogonia. Methods to transplant spermatogonial stem cells and germ line stem cells to seminiferous tubules in testes were used to demonstrate germ lineage commitment of the isolated cells (Brinster and Nagano 1998; Brinster and Zimmermann 1994; Kanatsu‐Shinohara et al. 2016; Kanatsu‐Shinohara et al. 2003), to study spermatogenesis, to study a variety of factors (nutrients, toxins, growth factors; (e.g., refs [Hakovirta et al. 1999; Nakayama et al. 1999; Oatley et al. 2009]) that can affect spermatogonia and spermatogenesis, and to provide a novel method for genetic manipulation (Kanatsu‐Shinohara et al. 2008; Kanatsu‐Shinohara et al. 2003; Ryu et al. 2007; Takashima and Shinohara 2018; Zhang et al. 2006). Many other studies found that the culture of spermatogenic cells of several different stages benefits from three‐dimensional matrices, coculture, and diverse other factors (e.g., (Choi et al. 2014; Ibtisham et al. 2021; Kanatsu‐Shinohara et al. 2003; Liu et al. 2024; Nagano et al. 2003; Nagao 1989; Takehashi et al. 2007)). Immortalized spermatogonial stem cells have also been used to achieve in vitro spermatogenesis (Feng et al. 2002). Additionally, very small embryonic like stem cells (VSELs, 1–3 μm) (Bhartiya et al. 2013; Parte et al. 2015), believed to be developmentally equivalent to late migratory PGCs, have been isolated from adult gonads and can restore spermatogenesis and fertility in mice when transplanted with Sertoli or mesenchymal stem cells (Bhartiya 2018; Parte et al. 2015). Spermatogonial cell transplantation to testis and in vitro production of sperm offer potential approaches to treat male infertility (Diao et al. 2022). The exciting progress and varied combinations of methods for culture and genetic manipulation continues to advance the field of in vitro spermatogenesis both for basic research and diverse applications in agriculture and medicine.

#### 2.1.2 Oogenesis

From the 1960s onward, oogenesis, oocyte physiology, and the biology of oocyte maturation and ovulation were studied in vitro using different approaches of increasing complexity and ability to sustain in vitro development. These included perfused mammalian ovaries (Lambertsen et al. 1976), cultured embryonic genital ridges (McLaren and Buehr 1990; Morohaku et al. 2016; Motohashi et al. 2004), cultured ovarian tissue pieces (Aakvaag 1970; Peluso and Hirschel 1988), isolated ovarian follicles (Kerin et al. 1976; Seamark et al. 1976), and isolated cumulus‐oocyte complexes (Nicosia and Mikhail 1975; Shea et al. 1975). These earlier studies provided a foundation for later efforts aimed at in vitro oocyte production. During the 1990s and thereafter, the development of oocytes from preantral stage ovarian follicles was described in several mammalian species using either ovarian tissue culture (Anckaert et al. 2009; Eppig 1996; Ghezelayagh et al. 2022; O'Brien et al. 2003; Peluso and Hirschel 1988; Telfer and Andersen 2021; Telfer et al. 2023; Wandji et al. 1997; Wandji et al. 1996) or reaggregated ovarian cells (Eppig and Wigglesworth 2000). Some methods included improvements in primordial follicle isolation (Dey et al. 2024) and use of mesenchymal cell coculture (Li et al. 2024). Increased interest in in vitro oogenesis from primordial follicles using fresh or cryopreserved ovarian tissue of humans or animals emerged as a possible means for restoring fertility or providing sources of oocytes subsequent to chemotherapy or radiation therapy in reproductive age women and childhood cancer patients. This has included studies to enhance survival and viability of oocytes using three dimensional extracellular matrix and other factors (Alkali et al. 2023; Herta et al. 2018; Hornick et al. 2012; Hossay et al. 2023; Laronda et al. 2014; Liu et al. 2024; Shea et al. 2014; Shikanov et al. 2011; Silber et al. 2022; Silva et al. 2016; Tagler et al. 2013; Tagler et al. 2014), ways to create “artificial ovaries” to enhance purity and avoid transferring cancer cells (Chen et al. 2022; Herta et al. 2018; Li et al. 2024; Morohaku et al. 2017; Morohaku et al. 2016), and studies addressing concerns about potential damage from tissue cryopreservation (Hossay et al. 2023).

Despite the above remarkable successes, gametogenesis purely in vitro from gonad‐derived cells and tissues has proven very challenging. Successes in generating mouse oocytes from fetal PGCs in vitro were reported with the use of an estrogen receptor antagonist (Morohaku et al. 2016) and additional modifications, such as controlling oxygen levels may further benefit the process (Tanimoto et al. 2022). Further major advances in IVG are emerging with the advent of stem cell‐based methods for producing primordial germ cells and gametes, potentially increasing the availability of in vitro derived gametes.

### 2.2 In Vitro Gametogenesis Using Isolated Stem Cells

The approaches for IVG using isolated stem cells leverage both older and more recent innovations for stem cell identification and isolation, induction of pluripotency in somatic cells, complex stem cell culture and differentiation systems, and either spontaneous or directed differentiation toward a gametogenic phenotype. Many of the methods applied during the pioneering studies using gonadal germ line cells have been applied in studies pursuing IVG with stem cells, along with additional innovations and advances. The array of sources of stem cells is highly varied and has expanded over the last decade.

#### 2.2.1 Testis‐Derived Stem Cells

There are significant challenges for IVG to derive sperm from stem cells, including the ability to achieve progression to spermatids and spermatozoa, as well as faithful execution of epigenetic changes, retention of genetic stability, development of culture systems to increase efficiency and gamete viability, and the ability of in vitro systems to support all of the necessary milestones of meiosis (Binsila et al. 2021; Lei et al. 2023; Samplaski et al. 2014; Sun et al. 2014; Tedesco et al. 2011; von Rohden et al. 2024). There is great interest and potential in using a variety of stem cell types to treat infertility, preserve fertility, or restore fertility after genotoxic or other injury (Gauthier‐Fisher et al. 2020; Lei et al. 2023).

In addition to PGCs, which can give rise to multipotent stem cells in vitro, the testis contains other stem cell‐like cells. Initial reports of pluripotent cell lines being derived from adult testis were called into question, as the expression of pluripotency marker genes, DNA methylation state at those genes, and an inability to form teratomas indicated that they differed greatly from authentic pluripotent embryonic stem (ES) cells (Tapia et al. 2011). Later analyses indicated a mesenchymal origin (rather than a germ cell origin), and a more restricted mesenchymal stem cell like state of these cells (Chikhovskaya et al. 2012; Chikhovskaya et al. 2014). Mesenchymal stem cells of various types can enhance spermatogenesis in vitro (Allam et al. 2025; Hsiao et al. 2019; Kadam et al. 2018; Önen et al. 2022) by creating a supportive spermatogenic niche (Chi et al. 2025; Gauthier‐Fisher et al. 2020; Liakath Ali et al. 2023; Liu et al. 2024; Peak et al. 2016). Stem cell derived extracellular vesicles have also shown promise for enhancing in vitro spermatogenesis (Liu et al. 2020; Taher et al. 2025). These reports suggest that non‐germ lineage testicular stem cells such as mesenchymal stem cells hold promise for supporting spermatogenesis rather than undergoing spermatogenesis. Additionally, the testis contains pluripotent VSELs that can give rise to spermatogenic cells (Bhartiya et al. 2013). Transplanting VSELs with companion Sertoli cells can facilitate this (Hong et al. 2022).

Another approach to deriving gametes from testicular cells has involved the generation of pluripotent or multipotent stem cells from germ line stem cells (Izadyar et al. 2008; Kimura and Nakano 2011). Subsequently, these stem cells can be used to generate spermatogenic cells or artificial testes in vitro.

#### 2.2.2 Ovary‐Derived Stem Cells

IVG to produce oocytes from stem cells must also satisfy the requirements of faithful execution of epigenetic changes, retention of genetic stability, supporting successful meiosis, as well as achieving both nuclear and cytoplasmic maturation required to support early embryogenesis. One possible source of stem cells for IVG to produce oocytes is the ovary itself.

The subject of germ line stem cells in post‐natal mammalian ovaries emerged as a controversial topic in 2004 and 2005 when Johnson et al. reported adult mouse ovaries could be repopulated with germ cells that form follicles (Johnson et al. 2004) and this could include an extragonadal source of such cells (Johnson et al. 2005). These reports challenged long‐standing dogma that female mammals could not replenish primordial follicles and that this was the basis for a finite reproductive span. The Johnson et al. studies were subsequently challenged (Byskov et al. 2005; Telfer et al. 2005) and there was much debate over technical aspects of the original studies and later studies seeking to confirm or refute them (Dunlop et al. 2014; Skaznik‐Wikiel et al. 2007).

After the initial reports of Johnson et al. other studies reported on the presence of female germ line stem cells (fGSCs) in ovarian surface epithelia in mice and human (Hu et al. 2012; Pacchiarotti et al. 2010; Parte et al. 2015; White et al. 2012; Zou et al. 2009). Other ovarian cells were also suggested as an alternate source of ovarian stem cells (Bukovsky et al. 2007). These included ovarian germ stem cells (“OSCs” or “OGSCs”; diameter about 4–7 μm) and the smaller VSELs (diameter about 1–3 μm), proposed to be the precursors of the larger OGSC stem cells in mice (Parte et al. 2015). On the basis of size and molecular markers the VSELs also appear to be distinct from, and may give rise to the ovarian stem cells reported by Tilly and co‐workers (5–8 μm) (Parte et al. 2015). VSELs can give rise to the OGSCs and form germ cell nests (Parte et al. 2015; Sharma and Bhartiya 2021). VSELs, which can also be isolated from other tissues such as bone marrow (Ratajczak et al. 2014) and have been isolated in multiple species (Dunlop et al. 2014) and by multiple laboratories (Ratajczak et al. 2019), are believed to be developmentally equivalent to late migratory PGCs and present within adult gonads (Bhartiya 2018; Bhartiya et al. 2013; Virant‐Klun 2018; Zuba‐Surma et al. 2009). They are quiescent but can be induced to proliferate in response to androgens and pituitary gonadotropins (Ratajczak et al. 2014), dividing asymmetrically to give rise to OGSCs, which are the ovarian equivalent of spermatogonial stem cells (Bhartiya 2015; Parte et al. 2015) and can undergo meiosis and differentiate as oocytes (Parte et al. 2015). As quiescent cells, mouse VSELs can survive chemotherapy drugs (Anand et al. 2016), but the niche required to support folliculogenesis and oogenesis is damaged by such treatments. Companion somatic cells such as mesenchymal stem cells can restore ovarian function in mice (Huang et al. 2019) and it may be possible to repair the niche (Hong et al. 2022). Additionally, fGSCs were reported to restore fertility in mice after damage caused by chemotherapy drugs (Xiong et al. 2015). It has been suggested that the termination of female mammal reproductive lifespan coincides not with the loss of a finite number of primordial follicles, but with the somatic cell senescence and loss of a suitable stem cell niche to allow VSELs and fGSCs to propagate (Bhartiya and Patel 2018). A variety of transplantation strategies, combinations of in vitro stem cell‐based methods, mesenchymal cell coculture “artificial ovary” systems, and three dimensional culture systems may also allow autologous fGSCs to be employed for reconstituting fertility following chemotherapy or aging (Hong et al. 2022; Martin et al. 2019; Xiong et al. 2015). This could include, for example, fGSC preservation and transplantation along with companion cells, or transplantation of somatic cells to restore the niche to allow endogenous fGSCs to proliferate and undergo neo‐oogenesis (Bhartiya and Patel 2018; Huang et al. 2019; Martin et al. 2019). What started as a controversial set of observations two decades ago may thus lead to new approaches to using stem cells in treating infertility.

#### 2.2.3 Gamete Derivation From Pluripotent Stem Cells

Significant progress and scientific discovery has in recent years accompanied the incorporation of pluripotent stem cells (PSCs) into the field of IVG. This has included the use of embryonic stem cells (ESCs), induced pluripotent stem cells (iPSCs), and conversion of germ line stem cells to a pluripotent state (Cui et al. 2023).

The methods for derivation of germ line stem cells from PSCs comprise an active and rapidly evolving field. Their design is driven by discoveries of major embryonic signaling pathways involved in establishing sequential states of developmental competency in the epiblast cells as well as anterior‐posterior patterning mechanisms (Schnirman et al. 2023). These pathways include bone morphogenetic protein (BMP), transforming growth factor beta (TGFβ), fibroblast growth factor (FGF), Wingless‐Related Integration Site family (WNT), and retinoic acid (RA). Preimplantation stage epiblast cells reside in a “naïve” state wherein they can contribute to diverse lineages including the germline, whereas postimplantation epiblast cells transition to a “primed” state wherein they can be induced for differentiation into other lineages (Schnirman et al. 2023). An intermediate state conducive to PGC formation is proposed to exist (Schnirman et al. 2023). Activin (TGFβ pathway) and FGF2 can promote the production of epiblast cells from ESCs, and the WNT pathway can promote or sustain the “naïve” state (Schnirman et al. 2023). WNT signaling appears to be necessary early in the process but WNT inhibition then becomes advantageous for primordial germ cell like cell (PGCLC) induction (Shono et al. 2023). GSK3 inhibitors promote WNT signaling by promoting accumulation of β‐catenin, subsequently promoting cell proliferation and inhibiting apoptosis (Chen et al. 2020). An optimum activity of WNT signaling appears to be required (Cantú et al. 2016; Kimura et al. 2006), as well as limiting the duration of WNT signaling, as WNT signaling is beneficial for early steps in PGCLC induction, but subsequent WNT inhibition is beneficial for PGCLC formation by preventing differentiation along other pathways (Sasaki et al. 2015; Shono et al. 2023; Vijayakumar et al. 2023). Consequently, treatment with low concentrations of Activin and FGF2 combined with WNT and RA signaling inhibitors may promote the intermediate state conducive to PGCLC formation (Schnirman et al. 2023). Signaling through the BMP pathway using BMP4 and BMP8a promote conversion to the PGCLC state (Schnirman et al. 2023).

**Figure 3:**
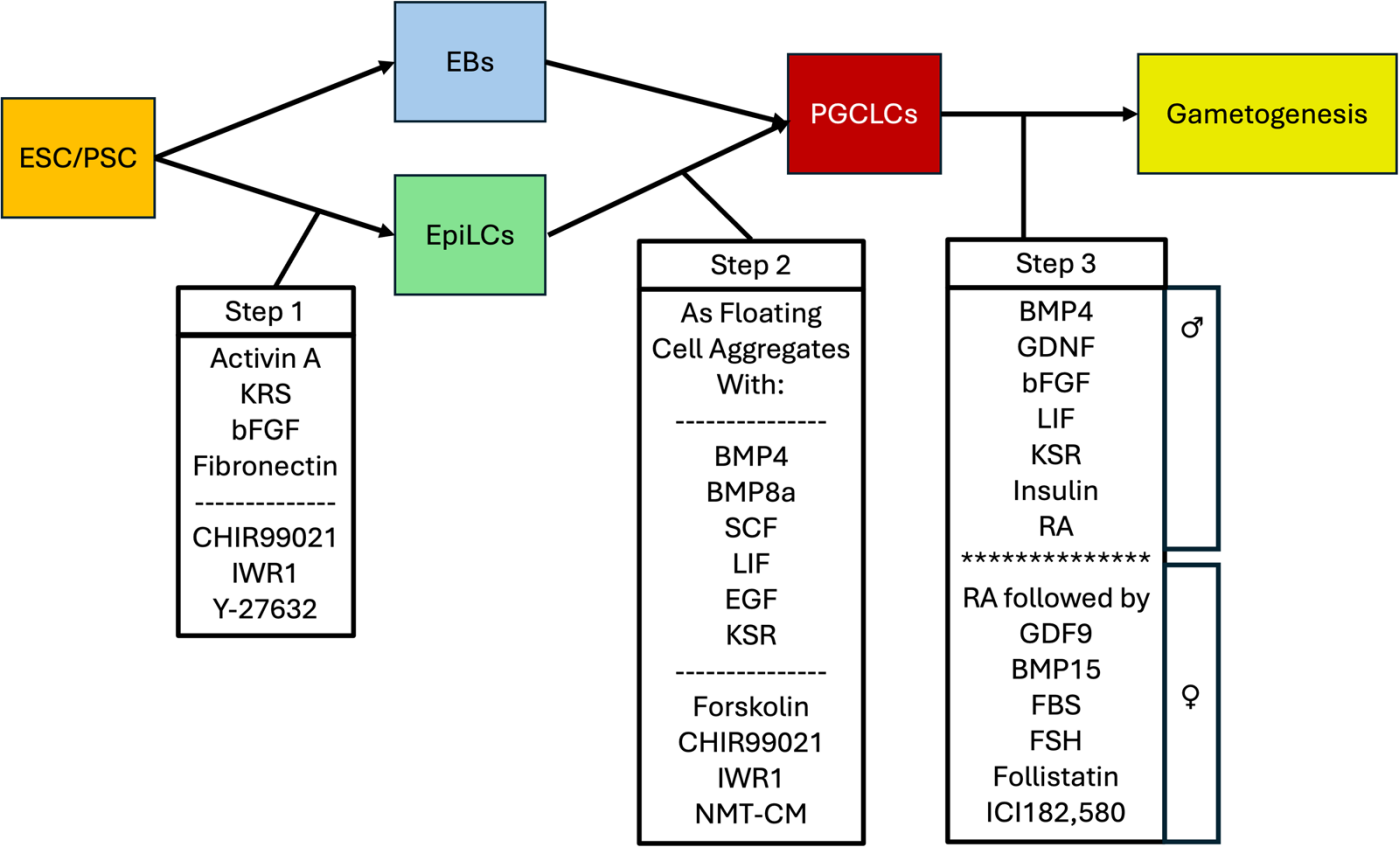
Examples of common factors employed for three major steps of IVG starting from ESCs/PSCs. The first step involves induction of ESCs/PSCs to epiblast‐like cells (EpiLCs) or incipient mesoderm like cells (iMeLCs), or the formation of embryoid bodies in suspension culture. PGCLCs can be isolated from the EBs or induced in a second step from EpiLCs or iMeLCs in floating cell aggregates using the factors indicated. PGCLC inducers can also be applied to embryoid bodies. The factors BMP4, BMP8a, SCF, LIF, EGF, and Knockout Serum Replacement (KSR) are common across multiple protocols and species, whereas forskolin, CHIR99021, IWR1, and NMT‐CM (neonatal mouse testis conditioned medium) differ more broadly by protocol and species. Factors employed for PGCLC induction in different species are summarized in Table 1. In the third step PGCLCs are induced to differentiate along male or female gametogenic pathways. This gametogenic phase is very complex and can be facilitated by many different system components, such as aggregation with companion somatic cells and sequential addition or subtraction of factors to mimic in vivo developmental progression.

To mimic PGC formation in the embryo, the process of generating PGCLCs from PSCs is thus often performed in two steps (Figure 3, Table 1). Either PSCs are first converted to embryoid bodies, or an early state variously termed epiblast‐like cells (EpiLCs), primitive streak‐like cells or incipient mesoderm‐like cells (iMeLCs) (Sasaki et al. 2015) either as a discrete step, or by applying a brief pretreatment followed by PGCLC induction. This first step employs WNT and TGFβ pathway activators. Factors such as Activin A, Knockout serum replacement reagent (KSR), basic fibroblast growth factor (bFGF) are common across species for this initial step (Table 1), with some protocols also employing ROCK (Rho Associated Coiled‐Coil Containing Protein Kinase 1), or GSK3 (Glycogen Synthase Kinase 3) inhibitors [e.g., cow, nonhuman primates, (Duggal et al. 2015; Sakai et al. 2020; Seita et al. 2023; Shirasawa et al. 2024; Shono et al. 2023)]. However, some protocols also employ inhibitors of WNT [e.g., cow, nonhuman primates, (Duggal et al. 2015; Sakai et al. 2020; Seita et al. 2023; Shirasawa et al. 2024; Shono et al. 2023)]. These treatments essentially make the cells competent to respond to subsequent PGC inducers whilst maintaining proliferation and inhibiting differentiation. The second common step in deriving PGCLCs employs PGCLC inducers to covert the cells from the above first step into PGCLCs. This second step commonly involves the use of bone morphogenetic proteins BMP4 and BMP8a, Stem Cell Factor (SCF), LIF Interleukin 6 Family Cytokine (LIF), and Epidermal Growth Factors (EGF) and some form of a low‐adherent or non‐adherent floating cell aggregate culture system (Aflatoonian et al. 2009; Cui et al. 2023; Hayashi et al. 2017; Hayashi and Saitou 2013; Ishikura et al. 2021; Kurek et al. 2020; Li et al. 2019; Seita et al. 2023; Shirasawa et al. 2024; Shono et al. 2023; Wang et al. 2016). PGCLC formation in species other than mouse may also employ some combination of other factors including forskolin, GSK3 inhibitor (CHIR99021), and ROCK inhibitor (Y‐27632), and retinoic acid [e.g, (Cheng et al. 2017; Sakai et al. 2020; Seita et al. 2023; Shirasawa et al. 2024; Shono et al. 2023)]. PGCLCs were induced in cattle ESCs using CHIR99021 and IWR1 in both pretreatment and PGCLC induction steps (Shirasawa et al. 2024). In some protocols, PGCLCs were induced from PSC in a single step [e.g., cynomolgus monkey, (Sakai et al. 2020)], and in some protocols, PGCLCs were derived from embryoid bodies with Activin, BMP4 or other factors provided [e.g., human, (Duggal et al. 2015)]. In addition to the above‐mentioned factors, many other factors have been incorporated into PGCLC derivation protocols in recent years (Kurek et al. 2020).

**Table 1:** Most common factors employed for deriving PGCLCs from ESCs and PSCs.

	EpiLC, EB/, or pretreatment	PGCLC induction
Mouse	Activin A, bFGF, KSR, Fibronectin	BMP4, BMP8a,^a^ SCF, LIF, EGF, FCAs
Human	EB, Activin A	BMP4, LIF, Forskolin, NMT‐CM, FCAs
Cow	BMP4, CHIR99021, Y‐27632, IWR1	BMP4, KSR, EGF, SCF, LIF, CHIR99021, IWR1, Y‐27632, FCAs
Pig	Activin A, bFGF, KSR	BMP4, BMP8a, LIF, SCF, EGF, FCAs
Cynomolgus Monkey	(single step process)	BMP4, LIF, SCF, EGF, Y‐27632, FCAs
Marmoset	KSR, Activin A, IWR1, Y‐27632	KSR, BMP4, SCF, LIF, Y‐27632, FCAs

Abbreviations: CHIR99021, GSK3 inhibitor; FCAs, use of low‐ or non‐adherent conditions for cultivating floating cell aggregates; IWR1, WNT inhibitor; KSR, knockout serum replacement; NMT‐CM, neonatal mouse testis conditioned medium; Y‐27632, ROCK inhibitor.

^a^ Change in name of reagent from BMP8b to BMP8a (Hayashi and Saitou 2013).

Additional strategies beyond the use of the above‐mentioned factors have been employed to derive PGCLCs. For example, mRNA transfection or genetic modifications to promote the expression of stimulating factors (e.g., SRY‐box Transcription Factor, SOX17; PR/SET Domain 1, PRDM1) have been employed in human and nonhuman primate cells (Irie et al. 2024; Kubiura‐Ichimaru et al. 2023). Additionally, many methodologies in different species have employed genetic modifications to provide for expression of fluorescent markers to facilitate visualization of conversion, or to allow enrichment for cells at steps in the overall process [e.g., (Hayashi and Saitou 2013; Ishikura et al. 2021; Sakai et al. 2020; Shirasawa et al. 2024; Toyooka et al. 2003)]. Such genetic modifications, while valuable to provide scientific insight into the process, could be undesirable for the reproductive applications of PGCLCs. Use of endogenous molecules for selection of coculture with cells producing inducing factors provide alternatives to direct genetic modifications in the PSCs. In some protocols, PGCLCs are obtained from embryoid bodies. An extensive review of various protocols for PGCLC induction for PSC and other cell types was recently provided (Cui et al. 2023). Despite commonality in some factors employed (Table 1), there is considerable variation in published protocols, particularly comparing protocols across species. This may reflect in part subtle differences amongst starting PSC populations with respect to sensitivity to the factors applied, fractions of cells converted to EpiLCs/primitive streak like/iMeLCs in the first step, and possibly the emergence of other cell types within the culture population that function as companion cells in supporting PGCLC formation. Comprehensive comparative studies could clarify the extent to which protocol variations reflect fundamental biological differences across species.

For the production of sperm or their equivalent from PSCs, ESCs can thus be induced to form PGCLCs in vitro either in embryoid bodies or in cell aggregates together with supporting cells. These can in turn be induced to form spermatogonium like cells, which can be propagated as germline stem cell‐like cells and eventually transplanted to form sperm and confer fertility (Geijsen et al. 2004; Nayernia et al. 2006; Saiti and Lacham‐Kaplan 2007; Toyooka et al. 2003). Inducing agents include retinoic acid and Glial Cell Derived Neurotrophic Factor (GDNF) (Li et al. 2019). Human ESCs cultured as embryoid bodies have been induced to generate PGCLCs with progression to post‐meiotic spermatids in vitro (Aflatoonian et al. 2009). Achieving complete male gametogenesis in vitro using defined factors rather than coculture conditions has been challenging. Mouse PGCLCs were induced to form spermatogonial stem cell like cells (SSCLCs) in vitro using a mixture of growth factors, a process that can be improved by overexpressing the Y chromosome‐linked gene *Eif2s3y* (Eukaryotic translation initiation factor 2, subunit 3) (Li et al. 2019). Subsequent differentiation to haploid spermatids in vitro was achieved with retinoic acid induction (Li et al. 2019). Human PSCs including iPSCs have been induced to form PGCLCs using a wide variety of culture protocols, including one‐step and multi‐step protocols, embryoid bodies, 3D culture conditions and use of extracellular matrices, and fetal gonadal cell coculture (Cui et al. 2023; Kurek et al. 2020; Leng et al. 2015). Roles for gonadal or other support cells either in priming PSCs before induction to PGCLSs, or for supporting PGCLC formation have been described in different in vitro systems (Hong et al. 2021). Studies to overcome early challenges with long‐term maintenance of human PGCLCs eventually led to culture conditions without feeders or serum (Kobayashi et al. 2022). In vitro male gametogenesis has been more challenging in livestock species, with early challenges in deriving PSCs (e.g., from embryonic disc or as expanded potential stem cells), which require FGF and WNT pathway signaling for maintenance (Botigelli et al. 2023; Goszczynski et al. 2023). Although livestock PGCLCs have been obtained, their long‐term culture and differentiation to functional gametes in vitro have yet to be achieved (Botigelli et al. 2023). But there is optimism in the field for the eventual success as species‐specific requirements for PSC and PGCLC culture are discovered and addressed (Botigelli et al. 2023), and indeed PGCLC derivation has been reported in both pig and cow (Shirasawa et al. 2024; Wang et al. 2016).

For the production of oocytes from PSCs, over two decades of study have yielded numerous exciting advances. For the earliest studies, mouse ESCs were cultured without feeders, and then cells expressing different combinations of a genetically modified germ cell‐specific Oct4‐green fluorescent protein reporter, *Kit*, *Vasa* and *Scp3* were isolated, representing premigratory or migratory germ cells, early post‐migratory germ cells, and post‐migratory germ cells ready to enter meiosis (Hubner et al. 2003). Cell aggregates that formed after 12 days in culture were collected and replated. About 20% of these aggregates formed ovarian follicle‐like structures producing estradiol and the oocyte factor growth differentiation factor 9 (GDF9). They also produced large oocyte‐like cells up to 130 micrometers in diameter having coats resembling zonae pellucidae and containing zona proteins ZP2 and ZP3 (Hubner et al. 2003). The oocyte‐like cells were spontaneously released after 26 additional days in culture or could be induced to be released with ovulatory stimuli (Hubner et al. 2003). Apparent parthenogenetic embryos developed in the cultures as well (Hubner et al. 2003). Culturing embryoid bodies with testicular cell conditioned medium as a source of growth factors also supported the development of presumptive oocytes (Lacham‐Kaplan et al. 2006). Treatment of PGCLCs derived from genetically modified mouse ESCs with retinoic acid (RA) and bone morphogenetic protein two (BMP2) promoted oocyte differentiation on feeder cells (Miyauchi et al. 2017). Another study derived mouse fetal ovarian somatic cell like cells (FOSCLCs) from ESCs and aggregated those with PGCLCs to generate developmentally competent oocytes in vitro (Yoshino et al. 2021). A culture system was established to allow follicle assembly and production of developmentally competent mouse oocytes (Morohaku et al. 2017; Morohaku et al. 2016). Transplantation of aggregates of PGCLCs together with somatic cells to the mouse ovarian bursa allowed the production of PSC‐derived oocytes (Hayashi and Saitou 2013) and PSCs were re‐derived from the resulting in vitro generated oocytes (Hikabe et al. 2016). A refined three‐step protocol with optimizations at each step was subsequently developed to allow complete oogenesis in vitro with the production of an increased number of oocytes in mice (Hayashi et al. 2017). The induction of PGCLCs to developing oocytes began with retinoic acid followed by an increasingly complex culture medium to support oogenesis, including a switch from GK15 to MEMα and from KSR to FBS, along with additional nutrients and other factors added in a specific sequence. These included follicle stimulating hormone, follistatin, Growth Differentiation Factor 9 (GDF9), Bone Morphogenetic Protein 15 (BMP15) and the estrogen inhibitor ICI182,580 along with female embryonic gonad somatic cells (Hayashi et al. 2017; Hayashi and Saitou 2013). But overall production efficiency remained lower than in vivo oogenesis, and the numbers of oocytes that could be produced was limited by availability of fetal ovarian cells to support folliculogenesis (Hayashi et al. 2017). Addressing other technical issues may increase success (Hayashi et al. 2017). The production of not only PGCLCs but also granulosa‐like cells from human iPSCs and aggregation with PGCLCs to form ovarian organoids in vitro (Pierson Smela et al. 2023) also suggested additional approaches to in vitro ovarian folliculogenesis and oocyte production using ovarian organoids derived from pluripotent cells.

The above successes in mice have been challenging to replicate in other species (Botigelli et al. 2023; Picton 2022). Studies with human and livestock species PSCs have shown the derivation of PGCLCs and the expression of germ line and oocyte markers, as well as the production of oocyte‐like cells that can form parthenogenetic embryos, production of ovarian somatic cell like cells, the use of two‐ and three‐dimensional culture systems, and production of follicles within reconstituted ovarian organoids (Botigelli et al. 2023; Wu et al. 2023; Yamashiro et al. 2018). The ability to produce oocytes as well as ovarian somatic cells from pluripotent cells combined with innovations in the culture systems has allowed much recent progress in in vitro oocyte production, but further advances are needed and anticipated (Bharti et al. 2020; Botigelli et al. 2023; Wu et al. 2023). These efforts are providing new insights into mammalian folliculogenesis and oogenesis, and potential new avenues for genetic engineering in animals and treating human female infertility.

#### 2.2.4 Other Sources of Stem Cells Used for In Vitro Gametogenesis

In addition to the ability to derive iPSCs from adult tissues, other types of stem cells can be isolated directly from tissues and can be used to generate PGCLCs. These include mesenchymal stem cells from bone marrow, Wharton's jelly or umbilical cord, and adipose tissue (Fayezi et al. 2022; Hu et al. 2015; Shlush et al. 2017), and stem cells from skin (Ge et al. 2016). However, the potential to form gametes may be limited (Ghasemzadeh‐Hasankolaei et al. 2018) compared to that observed for iPSC and PGCLCs, and PGCLC derivation is less efficient directly from these stem cells as opposed to using PSCs (Salvatore et al. 2023). Protocols for the induction of mesenchymal stem cells from human, mouse, goat and dog to form PGCLCs or other germ line cells have included the use of testicular cell conditioned medium, retinoic acid, testosterone, BMP4, BMP8a, Transforming growth factor beta (TGFβ1), or gene transfection to increase expression of other factors (Cui et al. 2023).

#### 2.2.5 Gamete Derivation From Genetically Manipulated Stem Cells

The above successes in in vitro gametogenesis paved the way for novel approaches incorporating the genetic manipulation of stem cells and in vitro production of genetically modified gametes and progeny. Recent studies provided striking demonstrations of these powerful new technologies to study genetic and epigenetic controls of gametogenesis, to correct genetic defects, to produce genetically altered animals, and to expand the applications of assisted reproduction.

A series of studies established that haploid ESCs derived from androgenetic or parthenogenetic embryos could be used as surrogate gametes for generating mouse progeny by nuclear transfer, albeit in some cases using genetic modifications of imprinted gene loci [(Aizawa et al. 2020; Li et al. 2012; Wan et al. 2013; Yang et al. 2012; Zhong et al. 2016; Zhong et al. 2015) and reviewed in (Aizawa et al. 2020; Bai et al. 2016)]. Diploid uniparental human ESCs were also derived (Ding et al. 2015). In one study, the roles of different imprinted genes in supporting development was explored by fusing haploid androgenetic and haploid parthenogenetic ESCs bearing different combinations of modified imprinted loci (Ma et al. 2025). These breakthroughs provided new capabilities for novel experimental studies including the introduction of genetic changes as well as other experimental treatments, such as exposure of cells to environmental agents or other stressors.

Two landmark studies yielded striking results that garnered broad international attention. These studies reported the production of bi‐paternal mice from genetically manipulated mouse ESCs.

The landmark study by Murakami et al. published in 2023, which produced bi‐paternal mice from genetically manipulated mouse ESCs, began with cultured male ESCs and identified a small fraction of colonies in which the Y chromosome had been spontaneously lost to create XO ESCs (Murakami et al. 2023). Subsequent culture of the XO ESCs employed a fluorescent marker to select for rare population of cells that underwent unequal X chromosome segregation and therefore converted to an XX genotype. PGCLCs derived from those neo‐XX ESCs were then used to develop oocytes in vitro using methods developed by Hikabe et al. (Hikabe et al. 2016). The conversion to the XX genotype could be slightly enhanced with the drug reversine. Conversion to the XX genotype was not accompanied by aneuploidy, and autosomal aneuploid ESCs were highly impaired in yielding PGCLCs capable of oogenesis. This alleviated concern about inducing autosomal aneuploidies during ESC conversion to the XX genotype, and showed that an aneuploidy could be corrected using a key portion of the ESC culture approach (Murakami et al. 2023). Importantly, oocytes derived from the neo‐XX ESCs could be fertilized with sperm to produce viable progeny, which were thus descended from two fathers.

Another landmark study was described by Li et al. in 2025, who induced genetic modifications in mouse ESCs (including frameshifts, deletions, and regulatory edits) at 20 imprinted gene loci (Li et al. 2025). This was achieved by genetically modifying androgenetic haploid ESCs and co‐injecting their nuclei with sperm into spindle‐depleted oocytes to yield androgenetic embryos in which half the genome contained the targeted imprinted loci. ESCs derived from those bi‐paternal embryos were tested for pluripotency by tetraploid complementation (Li et al. 2025). This study was preceded by one in which the authors generated viable and fertile bi‐maternal mice using targeting at three imprinted loci in ESCs and nonviable bi‐paternal progeny with targeting at just 7 imprinted loci (Li et al. 2018). Thus, progressively increasing the number of target imprinted loci to produce bi‐paternal mice increased viability and reduced developmental abnormalities, with targeting at 20 loci yielding viable and healthy male and female progeny (Li et al. 2025). However, limitations remain, because the gametes of bi‐paternal mice produced by genetically modifying 20 imprinted loci will contain thousands of different potential random subsets of those modified loci (Li et al. 2025). Female bi‐paternal mice were not fertile (Li et al. 2025). Male bi‐paternal mice produced viable sperm, but diploid bi‐paternal ESC lines derived from embryos produced by injection of bi‐paternal mouse sperm into oocytes together with haploid androgenetic ESCs failed to yield viable progeny (Li et al. 2025), indicating that the random segregation of subsets of the 20 modified loci results in lethality.

The results of studies of bi‐maternal mice prepared using ESC manipulations harken back to earlier nuclear transfer studies in which parthenogenetic mice were produced by chemically activating oocytes that contained one normal set of maternal chromosomes and one set from nongrowing oocytes (Kono et al. 2004). Those previous studies indicated that oocyte genomes in which imprinting had not been fully established could complement the defects of fully maternally imprinted genomes in supporting development. The more recent studies with bi‐paternal and bi‐maternal ESCs highlighted the additional practical advantages that they can be derived from adult cells, they can be genetically manipulated, different imprinted genes can be targeted to study imprinted gene biology, and genetic defects can be corrected, including aneuploidies. Using these ESC technologies in combination with other reproductive technologies thus provides a powerful new approach for studying the different roles of imprinted genes during development, the roles of other genes in many different developmental and physiological processes, and for studying the impact of genetic and environmental factors.

## 3 Perspectives—Importance, Challenges, and Ethical Considerations for the Future of IVG

### 3.1 Importance and Benefits of IVG

There is great potential value in using IVG systems both as research tools and in applications to treat or prevent infertility, promote agricultural animal breeding and herd improvement, or facilitate species conservation. The many remarkable experimental systems described above, and others, provide exciting new avenues for dissecting the underlying biology of imprinting, meiosis, germ cell‐somatic cell communication, and even early developmental mechanisms that rely on essential gametic inputs to support early embryogenesis and embryonic genome programming. Substantial insight has already been garnered from the use of haploid ESCs and the genetic manipulation of ESCs during the production of uniparental mice. These methodologies can be combined with other techniques to explore the roles of individual imprinted genes and combinations of imprinted genes in the elaboration of tissue phenotypes and functions, as well as how environmental factors may impact those genes and functions.

The ability to complete meiosis is recognized as a major barrier to IVG using many of the above systems (Sun et al. 2014), and available evidence suggests the role of essential germ cell‐somatic cell communication in enabling meiosis and supporting gametogenesis. Extensive manipulations of the IVG environment and discovering how such modifications impact meiosis may allow the mechanisms controlling the fidelity of meiosis to be discovered in detail. Additionally, IVG systems that support faithful recapitulation of essential processes key to gametogenesis, and that could be genetically, epigenetically or pharmacologically manipulated, could greatly accelerate our understanding of early determinants of embryo developmental potential.

A major limiting factor in many assisted reproduction settings is the limited availability of high‐quality gametes, particularly developmentally competent oocytes for use in the in vitro embryo production process applicable for improving herd genetics and expanding the availability of high genetic merit livestock (Vries and Kaniyamattam 2020). Despite the remarkable breakthroughs achieved to date, two clear limitations with some of the current IVG methods are a reliance of some methods on oocytes and constraints in efficiencies in the production of functional gametes in these systems. Overcoming these barriers to success may soon resolve long‐standing deficits in our understanding of what molecular characteristics allow high‐competence gametes to be formed. Enhancing the efficiency of formation of competent oocytes will by necessity reveal conditions and mechanisms that favor this outcome. In the process, IVG should become more efficient and improve outcomes in assisted reproduction techniques.

The emerging IVG methods, particularly those incorporating the use of stem cells that can be expanded and frozen for long‐term use, may also overcome concerns with gamete and gonadal tissue cryopreservation, such as oocyte viability and the need for surgical procedures. Additionally, more efficient methods of deriving PGCLCs, for example, from cryopreserved gonadal tissue, could provide additional options for the use of such tissue.

The ability to derive gametes from adult tissues and stem cells has numerous advantages, particularly in clinical fertility treatment. Cells can be isolated from a patient's own tissues, thereby providing immune compatibility in procedures involving stem cell transplantation. Concerns about how best to use a limited supply of a patient's own gametes can be avoided. There is also the exciting possibility of repairing genetic mutations and aneuploidies in established patient stem cell cultures, so that the derived gametes can be used with greatly reduced risk of producing human embryos bearing such genetic defects. The remarkable success of producing viable, fertile bi‐paternal or bi‐maternal mice makes possible broader application of assisted reproduction for same‐sex couples and illustrates the feasibility of deriving gametes from adult tissues for patients who otherwise lack a source of their own gametes. However, limitations remain, such as progeny infertility and other reported phenotypic abnormalities (Li et al. 2025).

### 3.2 Major Challenges Remaining for IVG

Significant barriers and challenges remain to be overcome for IVG, particularly for application in humans. The successful production of fully functional sperm entirely in vitro using isolated germ cells or stem cells has yet to be achieved. This barrier may be solvable by using spermatid nuclear injection, eliminating the need for mature spermatozoa. The overall inefficiency in completion of meiosis remains a major hurtle limiting the application of IVG. Differences in the epigenetic (e.g., histone modification and gene transcriptional states) and DNA methylation states of some stem cells, and gametes generated from them, compared to authentic gametes, requires further study. The overall efficiency of even the most recent and advanced IVG methods remains low, which will limit IVG application in large‐scale livestock production situations, and render IVG costly and inaccessible to many patients. Additionally, the lifespan of progeny produced with some IVG protocols is lessened, and other phenotypic differences have been reported (Li et al. 2025). These challenges and limitations likely reflect the need to more fully replicate the intimate cell‐cell interactions between somatic and germ lineage cells and the interdependence of developmental progression of both cell types during the normal process of gametogenesis. One approach to meeting that need could rely on transplantation methods. Transplantation of stem cell‐derived germ cells offers one possible approach, particularly for restoring spermatogenesis. Similarly, transplantation of stem cell‐derived somatic cells into ovaries to restore the germ cell niche could allow endogenous germ cells to initiate oogenesis at an early stage in the process. Other transplantation methods using cell aggregates may also be feasible. Fully in vitro methods for IVG yielding mature, functional gametes at high efficiency will require continued refinements and innovations in current methods. Improvements in three dimensional culture systems, the use of cell aggregates possibly combining multiple types of stem cell derivatives, treatments with growth, differentiation, or other exogenous factors, refinements in culture components to better fit the metabolic needs of gametogenic cells, and further development of sequential culture systems could all provide the necessary advances. Ultimately such refinements could solve many of the above major barriers. Such refinements will simultaneously rely upon and yield substantial improvements in our understanding of fundamental mechanisms of mammalian gametogenesis. Of further interest will be the discovery of the conserved or shared aspects of IVG in different species. While many common features are seen in protocols across species (Table 1), significant differences in reported protocols exist. This variation may reflect differences in the biology of germ cell formation and differentiation, but could also reflect a combination of differences in the starting material (PSC characteristics), differences in suitability of available reagents, differences in the use of genetic modifications to facilitate the process, and differences in methodologies that emerge as practitioner understanding improves over time. Increased knowledge of the shared or species‐specific pathways and mechanisms governing IVG is needed.

### 3.3 Ethical Concerns for Use of IVG in Humans

The major ethical concern facing the application of IVG technologies in humans is the assessment of the risk of birth and developmental defects and increased risk of diseases or disorders during later life for the progeny. The mouse model has seen the greatest success in IVG, especially in cases where genetic modifications and markers can be used to facilitate the process. Such modifications and markers would not be acceptable for IVG in humans. Despite the successes in mice, translation of outcomes to other species, even the rat model, has been challenging, and studies in other species have pointed to apparent species‐specific differences and requirements for IVG. This suggests that IVG would ideally be proven safe in multiple different species. This would require extensive testing for genetic and epigenetic equivalence of IVG gametes to naturally produced gametes, for the equivalence of genetic and epigenetic states of IVG progeny and naturally produced progeny, and for overall safety with respect to diverse metabolic and health parameters. Demonstrated success and safety in a nonhuman primate model would be especially valuable as a way to assess potential risks to the unique physiology, complex behavior and cognitive development and function that are central to human health and longevity. Applications of approaches for rescuing genetic defects must also be approached with caution and be carefully regulated, not only in humans but in other species as well, to avoid inadvertent negative impacts on population genetics. Cost and accessibility also comprise a significant ethical concern that will require a robust and safe methodology that can be applied routinely and made broadly available, an objective that may prove difficult to achieve.

### 3.4 Emergence of a Nondioecious Reproductive Mode for Humans?

While mammals naturally exhibit exclusively dioecious sexual reproduction, the above emerging IVG methods provide the capacity for *Homo sapiens* as a species to manifest an exception to this restriction. Both male and female gametes can be derived from either individual sex, or even from the same individual, expanding the array of available advanced reproductive technologies (e.g., cloning by somatic cell nuclear transfer) that circumvent normal mammalian reproduction. As such, the evolution of human intelligence and ensuing ability to devise ever increasingly complex technologies for modifying our environment and even journeying to other planets has now turned inward, and led our species to a new milestone, wherein a fundamental feature of mammalian biology can now be theoretically circumvented through multiple approaches.

## Author Contributions

**Keith E. Latham:** conceptualization, writing – original draft, writing – review and editing, visualization, funding acquisition, resources.

## Conflicts of Interest

The author declares no conflicts of interest.
